# Capturing Biomarkers and Molecular Targets in Cellular Landscapes From Dynamic Reaction Network Models and Machine Learning

**DOI:** 10.3389/fonc.2021.805592

**Published:** 2022-01-21

**Authors:** Susan D. Mertins

**Affiliations:** ^1^ Department of Science, Mount St. Mary’s University, Emmitsburg, MD, United States; ^2^ Biomedical Informatics and Data Science Directorate, Frederick National Laboratory for Cancer Research, Leidos Biomedical Research, Limited Liability Company (LLC), Frederick, MD, United States; ^3^ BioSystems Strategies, Limited Liability Company (LLC), Frederick, MD, United States

**Keywords:** biomarkers, molecular targets, drug discovery, drug development, pharmacodynamic modeling, ODE modeling, machine learning

## Abstract

Computational dynamic ODE models of cell function describing biochemical reactions have been created for decades, but on a small scale. Still, they have been highly effective in describing and predicting behaviors. For example, oscillatory phospho-ERK levels were predicted and confirmed in MAPK signaling encompassing both positive and negative feedback loops. These models typically were limited and not adapted to large datasets so commonly found today. But importantly, ODE models describe reaction networks in well-mixed systems representing the cell and can be simulated with ordinary differential equations that are solved deterministically. Stochastic solutions, which can account for noisy reaction networks, in some cases, also improve predictions. Today, dynamic ODE models rarely encompass an entire cell even though it might be expected that an upload of the large genomic, transcriptomic, and proteomic datasets may allow whole cell models. It is proposed here to combine output from simulated dynamic ODE models, completed with omics data, to discover both biomarkers in cancer *a priori* and molecular targets in the Machine Learning setting.

## Introduction

Understanding biological systems is challenging as the detail and complexity of such dynamic entities cannot be grasped using human ken and intuition. And disease states such as cancer further the difficulties in addressing living entities. Thus, investigators have created networks of cellular systems that encompass many components, connect those parts in some fashion, and then interrogate their usefulness for addressing predictability and/or to study the networks themselves (1). This has been well studied in the case of transcription factor networks that characterize the phenomenon of epithelial to mesenchymal transition (EMT) in cancer (2–6). Recent work demonstrated that the topology of transcription factor regulatory networks that were parameter free (using a Boolean approach) or were parameter agnostic (using a random parameter generator) was important in limiting changes in cell state that may promote disease progression (3). While important findings can be gleaned from understanding regulation of gene expression, the effectors are not included in such modeling nor are site-specific details such as binding affinity or catalytic activities such as phosphorylation that are likely to influence a particular cell behavior. Therefore, the remainder of the Mini Review will focus on the modeling approaches that include the depth and likely parameters that may improve useful predictions.

Modeling cell behaviors using the mathematics underpinning biochemical reactions has been a research topic for decades when first approached by Tyson and others in understanding the cell cycle in the 1980s (7). It was and still is clear that cellular networks and pathways are dynamic and the overall contribution to cell behavior mattered. Since then, a vast array of biological models described by ordinary and partial differential equations (ODE, PDE) have been published, new software has been created to entice bench scientists to advance their findings computationally [for example, RuleBender (8) and Virtual Cell (9)], and now, an unprecedented amount of data to supply those models is available. For the oncology field, melding both mathematical models and omics data holds the promise to select the most effective molecular targets and any concurrent biomarkers. The future promise consists of whole cell models that lead to decisions of personalized therapies needed to predict tumor regression.

At the cellular level, mathematical models describe reaction networks (e.g., signal transduction pathways and metabolic cycles) effectively, typically with output that is difficult to predict. For example, binding of a growth factor to its receptor is a reaction that is dependent on characteristics such as binding affinity and concentration and leads to downstream events of interest occurring with time. Further, if positive and/or negative feedback loops are considered, cellular function becomes less predictable by inspection, if at all. But, operationally, in this example, the change in concentration of bound and unbound receptors and growth factor with respect to time can be tracked through species in the ODEs and downstream effects delineated. Furthermore, properties such as molecular diffusion can be described with PDEs, thus including spatial considerations as well. Many other biological properties have been described and include cytoskeleton formation (10), vesicular transport (11), and gene expression (12). Hundreds of such mathematical models have been deposited in the BioModels database (13) (RIDD: SCR_001993).

It is the premise of this Mini Review to describe how computational dynamic ODE models describing biochemical reaction networks can be analyzed by Machine Learning (ML) algorithms capable of predicting desirable outcomes for the two of the present challenges in oncology: discovering biomarkers associated with positive patient outcomes and novel molecular targets not generally considered druggable.

## Selected ODE Models

As experimentalists have supplied an understanding of the essential knowledge of biochemical pathways underscoring cell behavior, ODE models offered predictions on some of the more interesting aspects. For example, stimulus response, in general, was thought to occur in a linear fashion such that the greater concentration of a growth factor, the greater the response. However, some components in pathways appeared not to follow this linear rule. Rather, an all-or-none response occurred. ODE modeling supplied a mechanism for such ultrasensitivity, demonstrating a phenomenon that occurs when proteins with enzymatic function are acting at saturation (14, 15). Other important discoveries ensued and included uncovering bistability, a behavior dependent on initial conditions. Bistability is defined as having two stable states at one stimulus level (16, 17). Parenthetically, bistability has also been defined to describe whole cell states such as those found in EMT and is distinct from that which is noted here (3–5). Functionally, bistability at the biochemical reaction level is of interest because, dependent on conditions such as saturation of an enzyme and high initial stimulus, cellular response can resemble a toggle switch in the on position. Thus, immediate downregulation may not be needed in these instances (16, 18). Finally, oscillatory behavior of important transcription factors such p53 and their regulators have been demonstrated through ODE modeling and in cells (19, 20). In addition, oscillatory levels of phosphorylated kinases have been characterized by an amplitude and frequency and were shown to be regulated and define outcomes such as the decision to proliferate (21). Below are two relevant examples of ODE models that can be exploited to discover biomarkers and molecular targets in oncology.

### MAPK Signaling

**Figure 1:**
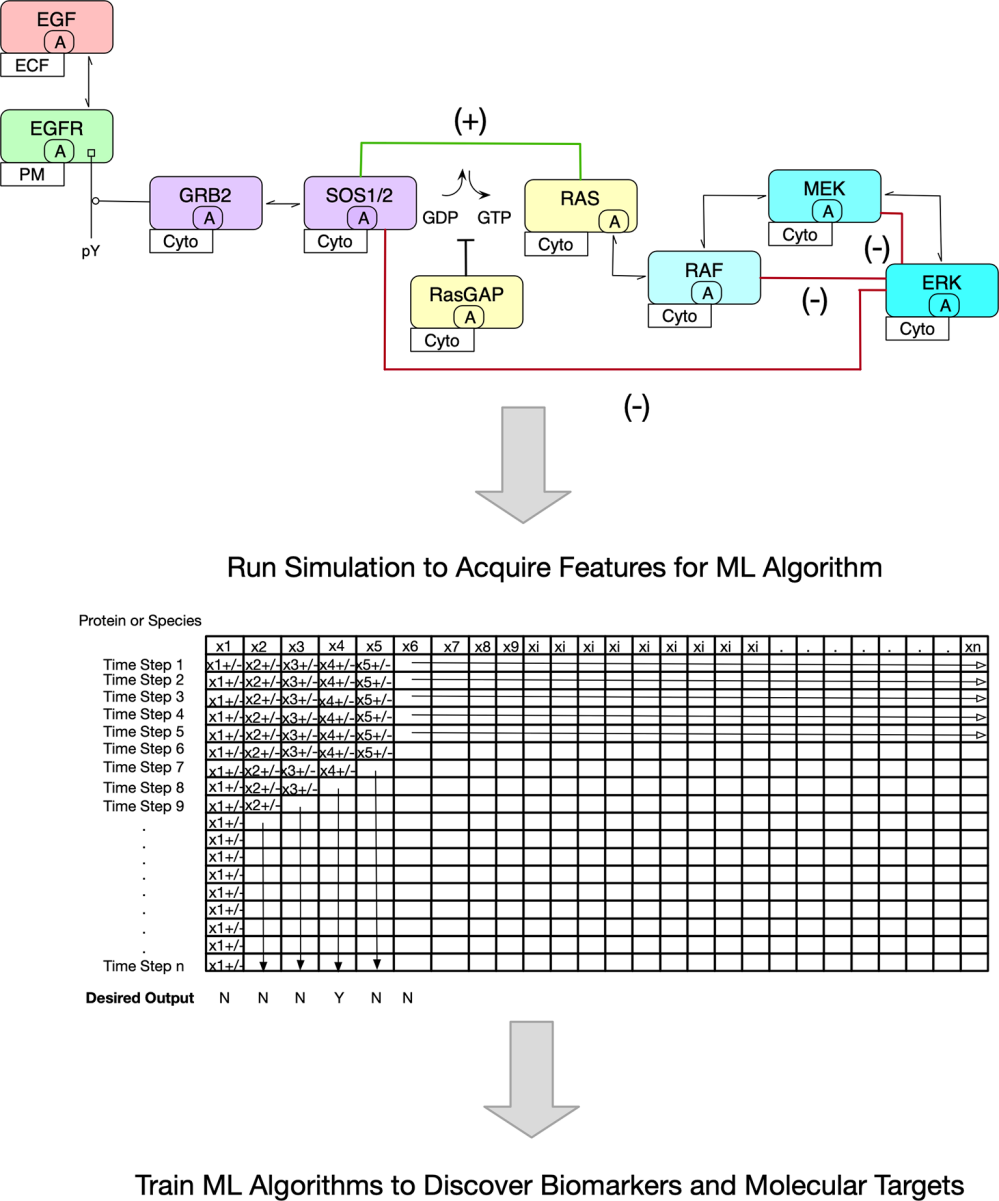
Overview of Proposed Methodology. The premise of this Mini Review is pictured. In the upper portion, a stylized version of the MAPK signaling pathway is shown. The contact map shown is the basis of an ODE Model. Note the inclusion of positive (green line) and negative (red lines) feedback loops. Because of these regulatory networks, it is inherently difficult to predict outcomes such as the decision to proliferate. Further, it is more challenging to ascertain pharmacologic interventions. In the lower portion, an abstract matrix is shown that depicts the changing protein concentrations with time once an ODE model is simulated. It is these data that are useful to train ML algorithms to discover biomarkers and novel molecular targets.

One of the most well-studied signal transduction pathways in oncology is that which triggers cell proliferation *via* growth factor stimulation. In one such reaction network, EGF, a growth factor, binds to its cognate receptor, which in turn, dimerizes and activates its kinase domain through conformational adjustments. Trans-autophosphorylation occurs next which forms the initial sites for adapter binding. GRB2 binding through its SH2 domain to phosphorylated tyrosine then binds SOS, a GTPase exchange factor. Critically, SOS replaces GDP with GTP on RAS, thus activating it for downstream binding of RAF, a kinase. A kinase cascade ensues ultimately leading to a phosphorylated kinase (phospho-ERK1/2) capable of triggering gene transcription necessary for cell growth. Importantly, several positive and negative feedback loops regulate the pathway and when considered in an ODE model, oscillatory behavior of phosphorylated ERK1/2 protein level occurs. While this description adequately corresponds to non-oncogenic signaling, disruption in the reaction rates by mutations leads to unpredictable outcomes dependent on pathway protein levels (22, 23). **Figure 1** (upper portion) depicts a limited Contact Map of MAPK signaling.

### Cell Cycle Arrest or Apoptosis Decision

Cell decisions regulated by p53 have been readily modeled as it is of deep interest to determine the conditions in which the outcome of cell cycle arrest or apoptosis occurs following genomic insult. This is of particular importance since p53 is commonly mutated in cancer and underpins tumorigenesis. Lipniacki’s group developed a sophisticated model wherein irradiation induced DNA damage, triggering p53 transcription and subsequent downstream reactions (24, 25). The p53 network was created using the rule-based modeling technique that defines biochemical interactions such as protein complex formation and enzymatic activities. In the three-stage model, a steady state was achieved first through simulation and solutions through deterministic algorithms. Next, irradiation was modeled over a range (1-10 Gy). In the last relaxation phase, cell behavior was characterized. Notably, the predicted outcomes were contingent on the degree of irradiation. Oscillatory behavior of p53 levels was observed as has been demonstrated *in vitro* and was found to define the conditions that lead to programmed cell death. Finally, their findings provided support for the heterogeneity in response to DNA damage observed in tumor cell lines. [N.B. For an overview of the methodology of ODE modeling in apoptosis, see (26)].

## Proteomic Databases

One of the challenges of ODE modeling is the need to insert quantitative parameters such as protein concentration in the reaction network. Parenthetically, precise measurements of reaction rates, enzymatic activity, and binding affinities are also critically important in computational modeling. The advent of critical technologies that can determine such include labeled and label-free assays such and SILAC and LC-MS/MS, respectively (27, 28). Both tumor cell lines and tumor tissue have been studied in this fashion and databases exist to exploit (29–32). And these quantitative proteomics efforts can evaluate post-translational modifications as well (33). The direct analysis of datasets from these studies has identified biomarkers, therapeutic targets, and drug resistance mechanisms. Further, and importantly, direct interaction networks can be constructed, but offer a static interpretation of a dynamic living cell.

The National Cancer Institute’s Office of Cancer Clinical Proteomics developed one such database that contains proteomic evaluations from 13 different tumor types collected since 2006 (31, 32, 34) (RIDD: SCR_017135). A series of studies merged both proteomic and genomic data to better classify actionable mutations that are expressed as proteins with certainty rather than through sequence alone. This is even more critical since transcriptomics does not necessarily confirm translation in as high as 50% of all transcripts measured. But importantly, studies utilizing this database have provided important new classifications in cancer histologies. One study evaluated the proteogenomics of pediatric brain tumors and found new subgroups with wild type BRAF and novel networks that overlap with the mutant gene (35). Thus, new therapeutic trials could be proposed for these challenging tumor types.

SILAC methods have been melded with mathematical modeling in the study of dynamic systems. For example, Yilmaz et al. investigated proteosomal processing of NFκB subunits in mouse embryonic fibroblasts *via* labeling studies and mathematical modeling to discover the dynamics of the system under activation (36). In another study, CHO cell extracts were processed in a glycoproteomic approach to understand N-glycan processing and found a kinetic description of the pathway (37). Finally, global protein synthesis rates were studied by pulse labeling and compared to mRNA synthesis rates in mathematical models, thus, providing critical quantitative parameters useful for future studies as well (38). In summary, SILAC and LC-MS/MS methods have supported mathematical modeling and hold further promise.

## Machine Learning

Applying ML algorithms to uncover cancer diagnoses from histologies, to determine therapeutic decisions, and predict outcomes is becoming pervasive in light of omics studies (39, 40). ML, in an overview, can sort through vast inputs and discover connections not likely to be found by human inspection. An alternative application for ML in oncology could include the development of hypotheses subject to future study. Thus, ML is suited to intake vast inputs and to realize relationships not previously expected. It is the thesis of this Mini Review to offer ways to meld ODE modeling and ML to discover biomarkers and molecular targets, ultimately aiding drug discovery and predictive oncology.

### Biomarker and Molecular Target Discovery

It is instructive to describe a published ODE model in more detail for EGFR signaling encompassing the MAPK pathway (**Figure 1**) to demonstrate how it might be utilized in ML with existing proteomic databases. In two models published by Creamer and colleagues (41) and Kochanczyk and colleagues (22), the basic signaling pathway is described for EGF binding to the family of cognate receptor tyrosine kinases with subsequent dimerization triggering autophosphorylation on their intracellular tails at multiple sites. Next, adaptors bind with some affinity *via* SH2 or PTB domains. In canonical MAPK signaling, bound GRB2 anchors SOS1/2, a GTPase exchange protein. During activation, SOS1/2 binds RAS and exchanges GDP for GTP, and so activates RAS. A kinase cascade follows RAF1 dimerization and binding to RAS. RAF1 phosphorylates MEK1/2 and it, in turn, phosphorylates ERK1/2 which translocates to the nucleus to modulate transcription regulating essential genes for cell proliferation. The ODE model of EGFR signaling as described here includes reaction rates that underlie binding affinities, turnover rates, phosphorylation and dephosphorylation rates, and both positive and negative feedback loops (22). Including these important regulatory features (the loops) is critical since unexpected behaviors emerge such oscillatory ERK1/2 phosphorylation levels and multiple states that include a steady state, a monostable one, or a bistable one dependent on the mathematical nature of the feedback loops. As an aside, even at this basic understanding, choosing a molecular target would be challenging.

Establishing the reaction network such as the one above by ODE equations can be readily accomplished with rule-based modeling using BioNetGen, a programming language and software that can further simulate the model over time (8). The simulations can be deterministically or stochastically solved with well accepted algorithms. Deterministic solutions are reproducible, resulting in the same output with every simulation. In contrast, stochastic simulations apply randomness to the solution, hence output is variable within a certain distribution and can be averaged. However, it is important to note that biological systems are noisy and the latter solutions may be more relevant. Simple output includes changes in species (molecule) abundance at each time step modeled. In the end, a vast amount of data is generated and fairly complex models with thousands of reactions can be coded (42). **Figure 1** (lower portion) pictorially describes a matrix of protein concentrations (in the columns) that change at each time step during a deterministic simulation (in the rows).

Parameterization of ODE models is challenging and typically, the scientific literature can provide many (43). It is anticipated that the proteomic databases described herein will readily provide protein abundance for ODE models and thus, may reflect both normal and disease states in separate models. Databases such as BRENDA (44, 45) (RIDD: SCR_002997) and Binding Database (46) contain curated reaction rates and affinities culled from the published literature. Thus, with a completed ODE model of interest, experimental protein abundance and measured parameters underlie their usefulness.

For ML algorithms, each time step can be represented by copy number (abundance) for an individual protein and thus, become features. What would be of interest for both biomarker and molecular target discovery is which state (i.e., time point and copy numbers) would be predictive of a desired outcome such as inhibition of cell proliferation and/or induction of cell death through one or more of the many known mechanisms. In order to achieve this, a training set would be needed that describes a signal transduction pathway or pathways anticipated to be central to cancer pathogenesis and clinically relevant. For example, the p53 ODE model such as the one described above intersects a critical cellular decision in light of DNA damage, determining cell cycle arrest or programmed cell death. The proteins in this training set model would be derived from tumor cell lines or tissues. Next a simulation would be completed resulting in a matrix of protein abundance at each time step (**Figure 1**). Now, the investigator has hundreds, if not thousands of models with and without the desired outcome that act as the training dataset for ML. The first analysis of such a ML model would be to find biomarkers (i.e., abundances of particular proteins) that correlate with the predicted outcome.

An alternative approach utilizing the same ODE model can aid in the discovery of novel molecular targets. In this case, proteins can be knocked out virtually (individually) through simple programming, a simulation run for each knock-out, and outcome collected. The ML algorithm would identify the connection between the presumptive molecular target and programmed cell death in this instance. Thus, a virtual high throughput screen has been completed using only computational effort.

## Conclusion

It has been proposed in this Mini Review to apply ML algorithms to discover biomarkers and molecular targets through the creation of ODE models of signaling pathways in cancer (47). While ODE modeling is more labor intensive than the ML analysis, the complex systems of cancer cell biology can be studied in this novel way leading to knowledge that will be readily apply to the pharmaceutical challenges ahead. In addition, ongoing advances in ODE modeling combined with tissue level simulations also show promise. For example, mathematical models of metabolism and cell proliferation indicated new molecular targets (48) and such multicellular models can further predict outcomes such as necrosis and growth arrest (49), cancer cell migration (50), and immune cell invasion (51). It can be envisioned that the computational efforts described herein can contribute to proposed Digital Twins for personalized medicine in cancer (52).

## Author Contributions

SM wrote the manuscript, completed the revisions, and approved of the submitted and revised version.

## Funding

SM was funded by Mount St. Mary’s University as Assistant Professor and BioSystems Strategies, LLC as Founder and CEO.

## Conflict of Interest

The author had a position as Guest Researcher at Frederick National Laboratory for Cancer Research, Leidos Biomedical Research, LLC, and is Founder and CEO of BioSystems Strategies, LLC.

## Publisher’s Note

All claims expressed in this article are solely those of the authors and do not necessarily represent those of their affiliated organizations, or those of the publisher, the editors and the reviewers. Any product that may be evaluated in this article, or claim that may be made by its manufacturer, is not guaranteed or endorsed by the publisher.
